# Efficiency and Productivity Change of Public Hospitals in Panama: Do Management Schemes Matter?

**DOI:** 10.3390/ijerph18168630

**Published:** 2021-08-15

**Authors:** José M. Cordero, Agustín García-García, Enrique Lau-Cortés, Cristina Polo

**Affiliations:** 1 Department of Economics, Universidad de Extremadura, Elvas s/n, 06006 Badajoz, Spain; jmcordero@unex.es (J.M.C.); cristinapf@unex.es (C.P.); 2 School of Medicine, University of Panama, Panama City 3366, Panama; elauc@css.gob.pa or

**Keywords:** hospitals, efficiency, productivity change, malmquist, DEA, management

## Abstract

In Latin American and Caribbean countries, the main concern of public health care managers has been traditionally placed on problems related to funding, payment mechanisms, and equity of access. However, more recently, there is a growing interest in improving the levels of efficiency and reducing costs in the provision of health services. In this paper we focus on measuring the technical efficiency and productivity change of public hospitals in Panama using bootstrapped Malmquist indices, which allows us to assess the statistical significance of changes in productivity, efficiency, and technology. Specifically, we are interested in comparing the performance of hospitals belonging to the two different management schemes coexisting in the country, the Social Security Fund (SSF) and the Ministry of Health (MoH). Our dataset includes data about 22 public hospitals (11 for each model) during the period between 2005 and 2015. The results showed that the productivity growth of hospitals belonging to the SSF has been much higher than that of the hospitals belonging to the Ministry of Health over the evaluated period (almost 4% compared to 1.5%, respectively). The main explanation for these divergences is the superior growth of technological change in the former hospitals, especially in the final years of the evaluated period.

## 1. Introduction

During the last decades, the main priorities of health policies in Latin America and the Caribbean (LAC) has been expanding health coverage and reducing health inequalities. Most of these countries have experienced great improvements in both areas thanks to the significant increase in public health expenditure (around 25% between 2000 and 2015), which is expected to continue in the future due to cost pressures arising from technological advances and growing and aging population [1]. This brings forward the need to promote policies that enhance efficiency of expenditures in the health sector, since efficiency gains can help contain future spending and contribute to raising the health status of the population.

This research focuses on the study of the health sector in one of those LAC countries, Panama, where the volume of budgetary resources allocated to the health sector has experienced strong growth in recent years (almost doubling in size between 2005 and 2015, whereas population only increased 20% in the same period). This increase in spending has been mainly due to the growth in salaries of healthcare personnel, the creation of new health facilities, and the large increase in budget allocations for medicines and medical and surgical equipment [2]. One of the most characteristic features of the public health structure in this country is its dual nature (i.e., there are two parallel financing systems or management schemes that coexist and provide health care services to the population): the Social Security Fund (SSF) and the Ministry of Health (MoH). This fragmentation in the financing of healthcare is relatively common in LAC countries, where there is usually one social health insurance scheme for the formal sector (SSF in this case) and a national health system (MoH in Panama) that guarantees coverage for the poor and those in the informal labor market [3]. The poor coordination between both systems has generated important distortions terms of the efficiency of the system due to the duplication of the costs of services provided, the asymmetry between the services offered, or the problems of inequity that arise between the urban and rural areas. Therefore, for years, there has been a strong interest in incorporating structural improvements in the model with the aim of reducing costs and avoiding inefficiencies. Unfortunately, so far it has not been possible to evaluate the performance of both management systems since there was not enough reliable information to make such an examination. Precisely the purpose of the present research is to shed some light on this issue by analyzing, for the first time, the efficiency and productivity levels of the Panamanian hospitals belonging to each system, since these institutions account for the largest proportion of the expenditure budget of both schemes.

Specifically, we examine the performance of a sample of 22 hospitals (11 belonging to the SSF and 11 belonging to the MoH) over an eleven-year period (2005–2015), so that we can explore which system has performed best over this period and how it has evolved over the years. To do this, it was necessary to collect a large volume of information on the Panamanian public hospitals, since the health system of this country did not have a formal register including data on the activities and services and available resources for these institutions, as is usual in most developed countries. Therefore, one additional contribution of this work has been the configuration of a database providing the basic information needed to assess hospitals, in which there is a unified and homogeneous register for each hospital and year studied.

In order to assess the operational efficiency of hospitals, we rely on a nonparametric approach which has been extensively used in empirical studies in the health sector [4,5,6]. Given that our database has a panel structure, we use a Malmquist productivity index (MPI) approach, which relies on the data envelopment analysis (DEA) in their calculation. This approach has been adopted in many previous studies to assess the managerial performance of hospitals [7,8,9,10,11]. This method allows one to determine whether changes in productivity over time are driven by changes in production technology, reflecting the changes of the production frontier between different periods, and changes in technical efficiency of the evaluated units in different periods (the so-called “catching up effect”). Likewise, if variable returns to scale are assumed [12], as in our study, it is possible to identify a third factor, represented by scale efficiency, which measures the degree to which hospitals gets closer to its most productive scale size over the periods under examination. In addition, we also apply the bootstrapping procedure proposed by Simar and Wilson [13,14] to accurately estimate the efficiency and productivity scores and confidence intervals that allow us to determine whether differences between estimates are statistically significant [15]. Despite the usefulness of this tool, there are still quite a few studies that have conducted statistical testing of estimators in DEA and Malmquist indices in the assessment of hospitals [16,17,18,19]. In this paper, we demonstrate that the results of traditional DEA and Malmquist index analyses need to be tested for statistical significance. Otherwise, the conclusions reached could be wrong.

The purpose of this study is to determine which of the two health financing systems that coexist in Panama (SSF and MoH) has performed better during the period studied and to identify the main driving factors behind this performance. In this sense, the results obtained may be very useful for policy decision making if health authorities of the country are interested in carrying out a restructuring process of the healthcare delivery and financing system, such as those implemented in other countries such as Brazil, Chile, Colombia, or Costa Rica where there was also an overlap between social health insurance and national health system schemes [3]. In this sense, the estimation of individual indicators for each hospital will allow us to identify the best and worst performers, which may help hospital administrators in benchmarking and establishing systems of rewards and/or penalties.

## 2. Literature Review

There is a vast literature on measuring the productivity and efficiency of health care institutions using both parametric and nonparametric approaches, especially for hospitals [20]. The main advantage of the former approach is that it allows for statistical testing of hypotheses about the production frontier and constructing confidence intervals around the estimated efficiency measures. Moreover, they also perform well with panel data since they take into account potential unobserved heterogeneity thanks to the use of econometric techniques. However, studies using this approach (for a literature review see [21]) require one to assume a certain functional form (usually with a single output) and a certain distribution for efficiency estimates, which is a major constraint. In contrast, the nonparametric methods are much more flexible, since do not require specifying any functional form that links inputs to outputs. In addition, they can easily handle multiple outputs in the transformation process, and they provide detailed information on areas of inefficiency. This explains why the majority of studies have opted for DEA to calculate efficiency scores as well as other closely instruments such as distance functions or Malmquist indices when panel data is available [22,23]. Likewise, in the most recent literature, it is increasingly common to find empirical studies evaluating the performance of hospitals by applying partial frontiers to mitigate the problem caused by the presence of outliers, extreme values, or noise in the data [24,25,26].

Within this literature, it is common to find studies that assess efficiency or productivity of different types of hospitals. In this regard, we can find a wide range of studies focused on analyzing the relationship between ownership and efficiency and, more specifically, comparing the performance of public and private hospitals [27,28,29,30,31,32,33,34,35,36]. Regarding this issue, the available evidence is mixed, but the public hospitals seem to be just as efficient as or more efficient than private hospitals, whereas private hospitals seem to be more responsive to (financial) incentives [37]. Another recurrent comparison is to analyze the performance of specialized versus non-specialized hospitals. In this case, the evidence is also mixed since some studies conclude that the efficiency levels of hospitals with specialization is lower [38], while other studies found specialization to be positively associated with technical efficiency [39,40].

Likewise, there is a stream of literature closely related to the main objective of the present research, which is mainly concern on examining the performance of public hospitals belonging to different financing systems [41,42,43,44,45]. For instance, Bannick and Ozcan [41] assess the performance of two branches of the US federal hospital system, the Department of Defense and the Department of Veterans’ Affairs, and conclude that the former outperform the latter. Similarly, Rego et al. [45] assessed the performance of traditional public hospitals and state-owned hospital enterprises in Portugal and concluded that the introduction of changes in the organizational structure of hospitals contributes to the achievement of higher levels of technical efficiency.

The vast majority of the aforementioned studies and, in general, most of the existing literature on measuring the performance of health care providers are referred to developed countries, mainly from Europe and North America [46]. Nevertheless, in the last two decades there has been a certain growth in the number of studies applied in low and middle-income countries [47,48,49,50]. Despite this, the available empirical evidence on Latin American and Caribbean countries is still very scarce. Among the main exceptions we can mention some empirical studies available for hospitals in Costa Rica [51], Brazil [52,53], Mexico [54], Colombia [55], or Ecuador [56]. Most of these studies analyze the effect of different reforms introduced in the health systems of these countries. The only one that focuses on analyzing the efficiency of hospitals belonging to different healthcare financing systems is the one referred to Mexico, where there are three different schemes (one private and two public). The results of this study suggest that public funding seems to be the best option for complex and high-technology hospitals, while privatization seems to be more efficient for smaller sized hospitals.

However, to the best of our knowledge, there is no previous evidence on Panama’s hospital system. Thus, the present paper constitutes an important contribution within this growing research area, since it compares the performance of Panamanian public hospitals operating under different financing and organizational systems for the first time.

## 3. Materials and Methods

### 3.1. Context of the Study and Sample Design

Panama is a Central American country with a population of approximately 4 million people, most of whom are concentrated in urban areas. The country is divided into ten provinces (Bocas del Toro, Chiriquí, Coclé, Colón, Darién, Herrera, Los Santos, Panama, West Panama, and Veraguas) with their respective local authorities and five comarcas. The public health sector serves 90% of the population through two healthcare providers, the Social Security Fund (SSF) and the Ministry of Health (MoH). The former is an institution that offers health care to the insured population (more than 85% of the inhabitants) and dependents through a network of comprehensive care services. On the other hand, the MoH has the mission of ensuring access to health services for the entire population and the whole territory, including rural areas with more difficult access.

The health system of the country is organized according to the degree of complexity of the services provided, distinguishing three basic levels of care. The first one is mainly composed of different typologies of primary care centers; the second level includes area and regional hospitals; and the third level is formed by national hospitals and national and supra-regional hospitals as well as several specialized hospitals on mental health, rehabilitation, and oncology. Our focus is on the upper two levels, since most of the health budget is concentrated in hospitals. In total, Panama’s public hospital network consists of 40 hospitals (26 belonging to the MoH and 14 to the SSF).

In order to conduct this research, it was necessary to build a database about Panamanian hospitals, since there was no formal register including data on the activities and services and available resources. This information had to be captured through a questionnaire designed specifically for the development of this research that was distributed to those responsible for the management of all public hospitals that are part of the system. The questionnaire included information on various performance indicators, budget indicators, available resources (physical and human), the center’s technological equipment, quality indicators, prevention programs developed, and data on patient management.

Since we were interested in capturing information over a sufficiently long period, it was necessary to provide the support of expert staff in statistics and medical records in some cases, since the existing data were widely dispersed. Therefore, one of the main contributions of this work has been precisely collecting the basic information needed to be able to carry out an assessment of the efficiency and productivity of these hospitals over an extended period. In this regard, we should note that the data collection process was far from simple, requiring a period of almost a full year to receive the completed questionnaire from a sufficient number of hospitals. In some cases, it was necessary to provide support from experts in statistics and medical records to organize the data, which, although recorded, were widely dispersed. Despite this, many questionnaires had significant deficiencies and limitations in the information provided, which forced us to discard some of the data requested, such as budget and quality indicators or data on the technological equipment available.

Although we would like to include in our analysis all the hospitals that are part of Panama’s public health system, we had to exclude some of them for different reasons. First, we decided to exclude six specialized hospitals to make the sample more homogeneous (e.g., the national institute of physical medicine and rehabilitation does not even have hospital beds). We were also forced to exclude four newly established hospitals from the analysis, since they had only been in operation for a number of years less than the period of analysis. Finally, hospital managers from eight hospitals did not report data about some relevant variables such as personnel or performance indicators, thus our sample is finally composed of 22 hospitals, whose names and main characteristics are shown in Table A1 included in the Appendix. It is worth mentioning that the hospitals included in the sample represent approximately 70% of total beds in the public hospital system and around 80% of the personnel. Thus we consider our sample to be representative of the total number of hospitals in the public health system.

### 3.2. Data and Variables

Our database includes data about eleven years (2005–2015), and thus the total sample consists of 242 observations (22 hospitals × 11 years = 242). The distribution of hospitals included in the sample between the two existing management models is equal (11 are part of the Ministry’s network and 11 belongs to the SSF). With regard to their geographical distribution, the sample includes information on most of the provinces (8 out of 10). The specific distribution of hospitals among the different provinces and management systems is displayed in Table 1.

Our selection of variables was based on previous hospital efficiency studies [5,23,57], but also taking into account the limitations of the data collected. As inputs, we selected the total number of beds as a proxy of capital and two variables representing human resources (medical and non-medical staff). As output variables, we use two quantitative indicators that are clearly linked to the intensity of resource consumption, such as the number of discharges and emergency services. Unfortunately, data about other potential variables representing hospitals’ outcomes employed in other empirical studies, such as inpatient rates, re-admissions or nosocomial infections, were available only for a limited number of hospitals.

Table 2 contains the main descriptive statistics for the whole sample (i.e., for the 242 observations available), and Table 3 reports these statistics distinguishing between hospitals under different financial systems. The high values of standard deviation shown in both tables reveal the existence of significant heterogeneity among hospitals, with very diverse sizes and wide variations in their resource endowment. It is also noteworthy that the number of beds is much higher in Ministry’s hospitals, although the volume of staff is slightly higher in centers belonging to the SSF. Likewise, the MoH´s hospitals clearly surpassed SSF´s ones in the two representative output variables. As expected, the average values recorded for emergency cases are clearly higher than those for discharges, since the former do not involve a process of hospitalization.

### 3.3. Methodology

Data envelopment analysis is a linear programming technique that allows for the development of an efficiency frontier based on input and output data from a sample of units. One of the major reasons for the use of this technique is its flexibility, since it does not require the definition of a set of formal properties that must be satisfied by the set of production possibilities. The aim of the data envelopment method is to build an envelope that includes all the efficient units, together with their linear combinations, leaving the rest of the (inefficient) units below it. The method provides a measure of the relative efficiency of organizational units considering simultaneously multiple inputs and outputs. Units located on the frontier have an efficiency score of 1 (or 100%), while the distance of the inefficient units from the envelope provides a measure of their level of inefficiency. The set of production possibilities estimated by DEA can be defined as follows:

(1) $$T=\left\{\left(x,y\right)\in R_{+}^{m}\times R_{+}^{s}:x\geq \overset{n}{\underset{j=1}{\sum }}\lambda_{j}x_{j},y\leq \overset{n}{\underset{j=1}{\sum }}\lambda_{j}y_{j},\overset{n}{\underset{j=1}{\sum }}\lambda_{j}=1,\lambda_{j}\geq 0,j=1,\ldots ,n\right\}.$$

This model implicitly assumes variable returns of scale (VRS) in production (the DEA model assuming constant returns of scale (CRS) is identical just without the convexity constraint). In other words, inefficient units are only compared with others that operate on the same scale. In this way, the technique is made more flexible by facilitating the analysis in those cases (very common) in which not all the units evaluated operate on a similar scale.

From the efficiency scores obtained with DEA, changes in productivity between two different time periods can be measured using the Malmquist Productivity Index (MPI). The formulation of this index was introduced by Caves et al. [58] and subsequently enhanced by Färe et al. [59]. Based on the concept of the output-oriented distance function [60], it can be defined as

(2) $$MPI_{C}^{t}\left(x^{t},\;y^{t},x^{t+1},y^{t+1}\right)=\frac{D_{C}^{t}\left(x^{t+1},y^{t+1}\right)}{D_{C}^{t}\left(x^{t},\;y^{t}\right)},$$

where the “*C*” suffix indicates that constant returns of scale are being considered.

Then, the geometric mean of the index can be defined by the following expression:

(3) MPICxt, yt,xt+1,yt+1=DCtxt+1,yt+1DCtxt, yt×DCt+1xt+1,yt+1DCt+1xt, yt12,

where MPI can take values greater than 1, which implies that there has been growth in productivity, values equal to 1, representing stagnation in productivity levels, or values less than 1, in which case the productivity of the units evaluated has declined with time. Furthermore, when using this approach, there are two main causes that can explain changes in the productivity levels of units: changes in technical efficiency (*EC*) (commonly known in the literature as the “catching up effect”), which indicates whether the units evaluated are moving closer or further away from their corresponding efficiency frontier between the periods evaluated and the technological change (*TC*), represented by the geometric mean of its magnitude, which approximates to what extent the units belonging the efficiency frontier have improved or worsened their productivity between the periods studied [22]. In the specific case of hospitals, the former can be interpreted as the improvements derived from the diffusion of best-practice technology in the management of hospitals and it is attributable to technical experience, management and organization, while the latter results from innovations and the adoption of new technologies by best-practice hospitals [61]. The most common decomposition of the Malmquist index is proposed by Färe et al. [59]:

(4) MPICxt, yt,xt+1,yt+1=DCt+1xt+1,yt+1DCtxt, yt×DCtxt+1,yt+1DCt+1xt+1, yt+1×DCtxt, ytDCt+1xt, yt12=ECxt, yt,xt+1,yt+1×TCxt, yt,xt+1,yt+1

This decomposition is based on the use of a production technology with constant returns of scale [62,63], through which we approach the notion of the average product. However, this definition can generate consistency problems when this assumption is not applicable, that is, if there are returns of scale in production [64]. Taking this argument as a reference, Färe et al. [65] redefined the efficiency change (*EC*) as:

(5) $$\begin{array}{cc} EC\left(x^{t},\;y^{t},x^{t+1},y^{t+1}\right) & =\left[\frac{D_{V}^{t+1}\left(x^{t+1},y^{t+1}\right)}{D_{V}^{t}\left(x^{t},\;y^{t}\right)}\right]\times \left[\frac{\frac{D_{C}^{t+1}\left(x^{t+1},y^{t+1}\right)}{D_{V}^{t+1}\left(x^{t+1},y^{t+1}\right)}}{\frac{D_{C}^{t}\left(x^{t},\;y^{t}\right)}{D_{V}^{t}\left(x^{t},\;y^{t}\right)}}\right]\\ & =PEC\left(x^{t},\;y^{t},x^{t+1},y^{t+1}\right)\times \frac{SE^{t+1}\left(x^{t+1},y^{t+1}\right)}{SE^{t}\left(x^{t},\;y^{t}\right)}\\ & =PEC\left(x^{t},\;y^{t},x^{t+1},y^{t+1}\right)\times SCA\left(x^{t},\;y^{t},x^{t+1},y^{t+1}\right) \end{array}$$

where *PEC* represents the change in pure technical efficiency and *SCA* the efficiency of scale (here, the “*V*” suffix indicates variable returns of scale). Thus, the Malmquist index can also be defined as:

(6) $$MPI\left(x^{t},\;y^{t},x^{t+1},y^{t+1}\right)=PEC\left(x^{t},\;y^{t},x^{t+1},y^{t+1}\right)\times SCA\left(x^{t},\;y^{t},x^{t+1},y^{t+1}\right)\times TC\left(x^{t},\;y^{t},x^{t+1},y^{t+1}\right)$$

Based on this decomposition, Ray and Desli [12] proposed a new decomposition of the Malmquist productivity index in which a frontier with variable returns of scale is used as a reference:

(7) $$MPI\left(x^{t},\;y^{t},x^{t+1},y^{t+1}\right)=PEC\left(x^{t},\;y^{t},x^{t+1},y^{t+1}\right)\times SCH\left(x^{t},\;y^{t},x^{t+1},y^{t+1}\right)\times PTC\left(x^{t},\;y^{t},x^{t+1},y^{t+1}\right),$$

where

(8) PTCxt, yt,xt+1,yt+1=DVtxt+1,yt+1DVt+1xt+1, yt+1×DVtxt,ytDVt+1xt, yt12

The change of scale factor can be broken down into the following terms:

(9) SCHxt, yt,xt+1,yt+1=DCtxt+1,yt+1DVtxt+1,yt+1DCtxt, ytDVtxt, yt×DCt+1xt+1,yt+1DVt+1xt+1,yt+1DCt+1xt, ytDVt+1xt, yt12=SEtxt+1,yt+1SEtxt, yt×SEt+1xt+1,yt+1SEt+1xt, yt12.

The change of scale efficiency component of the above equation is the geometric mean of two measures of change of scale efficiency. The first is defined with respect to the technology of period t and the second with respect to the technology of period *t* + 1.

To calculate efficiency scores, productivity indices and the different components we rely on DEA. With the aim of improving the accuracy of the estimations, we obtain confidence intervals for the different components of productivity by applying bootstrap procedures, thus we can make reliable statements concerning whether they are significant. Bootstrapping, introduced by Efron [66] and Efron and Tibshirani [67], has proven effective in examining the sensitivity of efficiency and productivity measures to sampling variation. This method is based on the assumption that for a sample of observations with an unknown data generating process (DGP), the DGP can be estimated by generating a bootstrap sample from which parameters of interest can be derived. The process involves using the original sample to construct an empirical distribution of the relevant variables by sampling the original data set repeatedly generating an appropriately large number (*B*) of pseudo-samples (in this study the process has been repeated 1000 times to ensure adequate coverage of confidence intervals). Then, relevant statistics such as means or standard deviations can be calculated by applying the original estimation process to the re-sampled data. Once we have a large and consistent estimator of the DGP, the bootstrap distribution will imitate the original sampling distribution. This implies that we can estimate the bias of each estimator, the bias corrected estimator and confidence intervals [68].

Regarding the estimation of confidence intervals for MIP, we use the smoothed bootstrap approach suggested by Simar and Wilson [13], which provides more accurate estimations than the traditional naïve bootstrap [67]. If the obtained bootstrapped confidence intervals do not include the number one, then the estimated MIP statistically significantly differs from unity, and therefore it is possible to be sure that there has been productivity growth (if smaller than 0) or deterioration (if greater than 0) is indicated.

## 4. Results

This section shows the main results obtained after applying the methodologies presented in Section 2. The procedure followed for the estimation of the productivity indexes presented below is based on an inter-temporal analysis considering each pair of years, thus calculating ten different Malmquist indices. The first would reflect productivity between 2005 and 2006, the second between 2006 and 2007, and so on until the index reflecting the change between 2014 and 2015 is reached. Therefore, when analyzing the evolution of productivity in the period assessed, what is being represented is the average value of these ten indexes and not the index calculated between the first and the last year.

Table 4 provides a summary of the results for the whole sample of hospitals, including the descriptive statistics (mean, standard deviation, maximum and minimum) of MPI (second column) and their different components for the period 2005–2015. The third column shows the estimated values for the evolution of technical efficiency (“catch-up” effect), which can be decomposed into two components, pure efficiency and changes in scale (fourth and fifth columns). The sixth and following columns present the other component of the MPI, technological change, and its decomposition into pure technological change and scale variations.

As can be seen, the Malmquist productivity index has an average value of 1.0253, which indicates that the productivity of Panamanian hospitals experienced an average growth of around 2.5% during the period between 2005 and 2015. This increase is mainly explained by technological change, while efficiency has hardly increased during the period.

The bootstrap sample means offer further insight into the results discussed above. Table 5 shows the mean 95% confidence intervals of MPI and its main components (EC and TC) for all hospitals and time periods, which were derived through bootstrapping as described in the methodology section. The interpretation of the confidence intervals is straightforward. Since all of them contain unity, it is not possible to statistically conclude whether there is growth or deterioration. This demonstrates that we should be cautious when analyzing mean results from the original sample.

**Figure 1:**
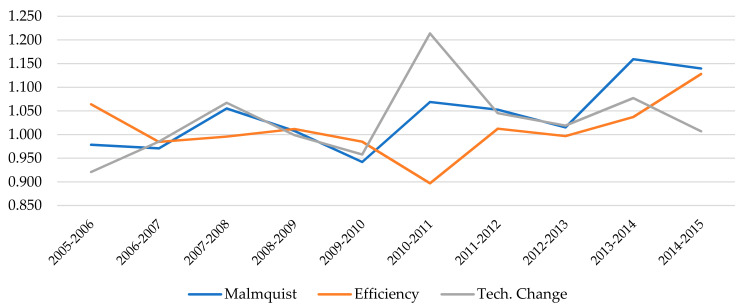
Evolution of the Malmquist Index and its main components (2005–2015).

Figure 1 shows a graphic representation of the evolution experienced by each of the main components (efficiency and technological change) throughout the period between 2005 and 2015. Additionally, in the Table A2 included in the Appendix, we also report the specific values of MPI and its components for each of the different years under evaluation. As mentioned above, the evolution of productivity is fundamentally linked to technological change. Both values follow an upward trend until 2008 and, after a small decline, they experienced remarkable growth in the early years of the new decade. The explanation for this result can be found in the investments made by several hospitals in these years with the aim of improving their infrastructures, which contributed to shifting the production frontier. However, in the last years of the period there is a certain divergence between them, as the MPI grows driven by EC, while the TC remains almost constant. It is also worth noting that efficiency change presents values very close to the unit in the first years and, subsequently, falls significantly until 2011, when it starts a continuous growth until the end of the period. These improvements registered in technical efficiency denote upgraded organizational factors associated with the use of inputs to be able to increase the level of outputs.

If we focus on the comparison between the two management models, the descriptive statistics reported in Table 6 show that hospitals belonging to the SSF outperform MoH´s hospitals throughout the period studied according to the MPI values. We can also see that this advantage is mainly due to technological changes, since average efficiency growth has been very low in both systems. In contrast, the average growth recorded by the SSF hospitals in TC is twice the average increase registered by the Ministry’s hospitals, whose growth is explained solely by factors of scale, while in the SSF hospitals it is attributable to both scale and pure technological change.

Again, the values of the confidence intervals of MPI and its main components (EC and TC) for bootstrap sample reported in Table 7 allow us to be more precise in the interpretation of results. The MIP of hospitals belonging to SSF shows that there was significant progress at the 5% level, as the confidence interval ranges from 1.0008 to 1.0919. Nevertheless, the confidence intervals for EC and TC for this group of hospitals both include unity. Therefore, it is not possible to statistically conclude that the MPI growth experienced by those hospitals can be attributed to technological change. As for the Ministry´s hospitals, all intervals contain unity, and thus we cannot make statements about whether they experienced growth or not during the period.

**Figure 2:**
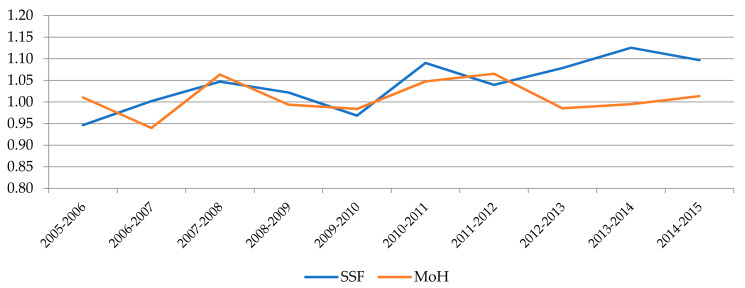
Evolution of MPI by type of management.

**Figure 3:**
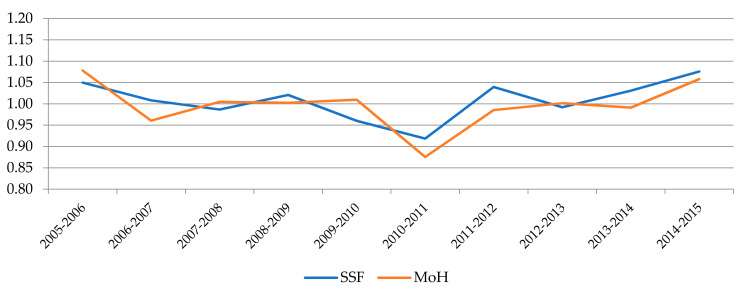
Evolution of EC by type of management.

**Figure 4:**
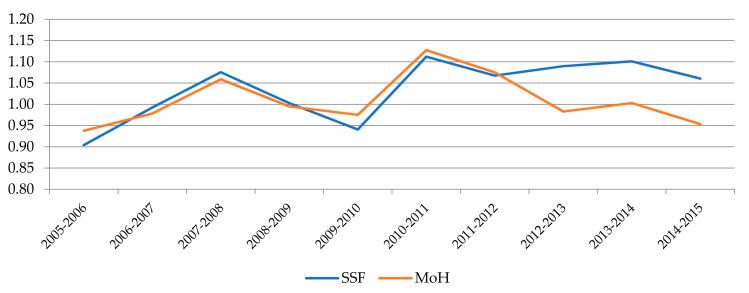
Evolution of TC by type of management.

Figure 2, Figure 3 and Figure 4 show the evolution experienced by the productivity indexes and their main components (EC and TC) over the years, distinguishing between hospitals belonging to each financing model. In Figure 2 we can notice that the evolution of MPI shows a similar trend for both systems until 2012, when a large gap opened between them as a result of the greater growth experienced by SSF hospitals, while the Ministry´s hospitals suffer a slight drop followed by some stagnation. The main explanation for these divergences observed in the last years of the period can be found in the evolution of technological change (TC), as shown in Figure 4. The analysis of the evolution of the components also allows us to appreciate that the efficiency change (EC) had a negative trend in both models until 2011. Since then, there has been a notable growth in both as well, although it has reached a higher level in SSF hospitals.

Finally, in Table 8 we provide the mean values of MPI and their components for each hospital throughout the whole period. Moreover, the confidence intervals estimated through bootstrapping for MPI, EC, and TC in order to account for statistical significance are also presented. Since hospitals are ranked according to the average MPI growth experienced over these years, we can notice that seven of the first nine belong to the SSF, while most of the Ministry’s hospitals are in the middle and lower part of the classification. If we focus on hospitals with a productivity index higher than one and examine their components, we find cases in which technological change is more relevant (e.g., Almirante, Horacio Díaz Gómez, or José Domingo de Obaldía), but also others in which efficiency change prevails (Azuero Anita Moreno, Changuinola, San Miguel Arcángel, or Gustavo Nelson Collado). Nevertheless, as for the hospitals with lower average MPI values (Joaquín Pablo Franco, Ezequiel Abadía, and Cecilio A. Astillero), all of them show higher values in the EC component, which may indicate that these hospitals have made less effort in terms of investment.

With respect to the values of the confidence intervals, in the nine hospitals with a higher MPI score we observe that almost all present lower values are above one, so we can consider these estimates to be sufficiently robust. For the rest of hospitals, the estimated intervals include the value one, which implies that the estimates obtained are not significant. This problem is much greater if we look at the estimated values for each of the components in which the oscillation is much greater. Thus, it is impossible to find an estimate that can be considered as significant. Therefore, as mentioned earlier, we need to be cautious when interpreting the results derived from the average decompositions.

## 5. Discussion

The main questions we attempt to address in this paper are determining how productivity and efficiency of the Panamanian hospitals have evolved in recent years and whether there are divergences between hospitals belonging to the two management systems that coexist in the country (SSF and MoH). Using data from 2005 through 2015, we applied a Malmquist productivity index and, subsequently, we employ a consistent bootstrap estimation procedure for correcting and obtaining confidence intervals for our estimates.

Regarding the first question, our empirical analysis has revealed that Panamanian public hospitals experienced a slight improvement in their productivity levels (2.5%) throughout the eleven-year period evaluated. Separating productivity growth into “catching up” (the less efficient hospitals improving) and “technological change” (the production frontier shifting outwards) may give important information for policy makers. However, the results presented here fail to provide a clear picture. Initially, we identify that the growth in productivity is mainly explained by technological change, while there has been hardly any change in efficiency levels, but the bootstrap estimates for the whole sample of hospitals were not statistically significant. Thus it is difficult to derive plausible policy explanations.

Despite this, we can nevertheless draw some insights regarding the second question. Specifically, we observe that hospitals belonging to the SSF have experienced significantly higher productivity growth than MoH’s hospitals, which has been particularly evident in the last years of the evaluated period. We also observed that this improvement has been driven by technological change, although again the bootstrap estimates are not statistically significant. Another interesting finding is that a large part of this technological change is due to scale efficiency effects (i.e., the larger hospitals with a wider range of technological equipment are the ones that have increased their efficiency levels the most as a result of a greater investment effort to improve their resource endowment and equipment). This finding is also relevant and in line with those obtained by Giménez et al. [54] for Mexican hospitals, although their results referred to a single year, and thus they were not able to analyze the behavior of different types of hospitals over time.

Although these results are very suggestive, it would be necessary to have more extensive information on the specific reforms implemented by hospitals in order to gain a deeper understanding of the origin of the improvements (or worsening) in terms of efficiency and productivity achieved during the period. Thus, for example, several studies have shown that increasing investment on health information technology may increase hospital productivity [69,70]. Unfortunately, we do not have information on the level of digitization available to each hospital, although we suspect that it is relatively low given the difficulties we experienced in the data collection process, so an in-depth study of this issue is beyond the scope of this study. In the same way, we are also unaware that during the years evaluated the Panamanian health authorities have introduced some specific reforms aimed at improving the productivity of the health system, so we cannot determine whether our findings are derived from a concrete reform. In this sense, it is worth mentioning that in recent years many countries have implemented reforms marked by financial restructuring of their health systems (increasing concentration by allowing mergers of hospitals) to enhance productivity, solve equity problems and facilitate access to health services. Therefore, it is becoming increasingly common to find studies that use frontier techniques such as DEA or MPI to evaluate the impact of those reforms [52,56,71,72,73]. Therefore, the methodology proposed in this study could also be applied for this purpose in the event that the Panamanian authorities decide to implement some specific reforms regarding the structure of its health system in the near future.

Finally, we should also mention that we have not been able to include in our empirical analysis information about some variables that may be relevant in the production process of hospitals due to the lack of reliable data. These include the use of some indicator of the quality of health services or some type of complexity-adjustment (e.g., case-mix index). Unfortunately, this is a common problem in studies conducted in developing countries [74], that needs to be addressed as information collection systems and the computerization of medical records are improved in all countries thanks to the increasingly frequent incorporation of new technologies in hospital management.

## 6. Conclusions

Hospital productivity measurement is an ongoing challenge in the research agenda of health care systems worldwide, since it provides to the manager/policy makers with an initial evaluation tool to compare the situation of each hospital with other similar hospitals and also look for benchmarks that can be used to design improvement strategies. In particular, the Malmquist productivity index based on data envelopment analysis has been proved to be very useful in assessing the performance of these organizations in very different frameworks [75,76,77]. In this study, we contribute to the existing research on this field of research by applying those methods to assess the performance of public hospitals in Panama, a country where no previous study had analyzed the performance of these entities so far.

The main focus of our analysis has been placed on the comparison between the two co-existing public management system, the Social Security Fund (SSF) and the Ministry of Health (MoH). This dual structure is relatively common in Central American countries, where there is a social insurance system offering health care to the insured population and a national health system that guarantees coverage for the entire population and the whole territory including areas with more difficult access. Our results suggest that hospitals belonging to the SSF outperformed Ministry´s hospitals throughout the period studied. According to the values of the Malmquist indices, the former experienced, on average, a productivity growth close to 4% during the period analyzed, while the Ministry´s hospitals only registered an increase of 1.5%. Moreover, the estimated confidence intervals confirm that the growth of productivity experienced by hospitals belonging to the SSF was significantly higher. Actually, if we examine specific hospital cases, we observe that eight hospitals show significant progress in MPI over the period, six of which belong to SSF.

The main factor that, in principle, explains these results is the superior growth of technological change in SSF hospitals, especially in the final years of the period. This result is not surprising, since the hospitals belonging to the Social Security Fund made a greater investment effort to improve their resource endowment during the period. However, it is not possible to statistically conclude that the MPI growth experienced by those hospitals can be attributed to technological change because the confidence intervals of this component, as well as for the efficiency change, are very wide and include the unity. Therefore, we must be very cautious in interpreting this result. In particular, our results reveal that we need to be careful when solely considering results from the original sample, especially if the sample size is not too large as in our case, without making statistical inferences (e.g., using bootstrapping techniques). This is something that has been already pointed out in some previous studies conducted in other fields of research [78], which is becoming an increasingly common practice when analyzing the efficiency of hospitals [10,19].

Despite the interesting findings derived from this empirical study, we are aware that it can still be improved and extended in several directions. First, we need to increase the number of hospitals included in the sample in future studies so that the results obtained may reflect in the most reliable way possible the reality of the country’s public hospital system. In this regard, it should be noted that there is great interest on the part of the Panamanian health authorities in implementing a computerized record-keeping system that will make it possible to capture information on the main resources and results of public hospitals, which would make it possible to continue and expand research in this field.

Second, in order to obtain results that more clearly reflect the differences between the two management models, we would have liked to be able to perform a meta-frontier analysis, as used in other studies comparing different management models [35,54]. Unfortunately, the small size of our sample, consisting of only 11 hospitals belonging to each management system, led us to discard this option since we would be below the empirical threshold levels for which the discriminatory power of the nonparametric would be very weak due to the so-called “curse of dimensionality” [79,80].

Third, it should be necessary to make an additional effort to collect data about several indicators that are frequently included in empirical study, such as the case mix of patients, so that we can determine the severity of cases treated in each hospital as well as the quality of the services provided. The consideration of these factors can be a relevant issue according to the results obtained by Chowdhury et al. [10].

Finally, it would also be desirable to consider some contextual or environmental factors that could influence (positively or negatively) the performance of hospitals. These could be socioeconomic and demographic variables, such as the percentage of people above 65 years old or the morbidity rate of the population living in their area of influence. These issues were not considered in the present study but should be addressed in a further analysis using a two-stage bootstrap approach to enrich the reliability of the results [81,82].

## Figures and Tables

**Table 1:** Distribution by province and financial system of the hospitals in the sample.

Province	Total	SSF	MoH
Bocas del Toro	3	3	0
Chiriquí	2	1	1
Coclé	2	1	1
Darién	1	0	1
Herrera	2	1	1
Los Santos	3	0	3
Panama	6	3	3
Veraguas	3	2	1
Total	22	11	11

**Table 2:** Descriptive statistics for the pooled sample with all observations.

Variables		Mean	SD	Min	Max
Outputs	Discharges	8885	8598	407	32,009
	Emergencies	52,153	36,082	2717	171,744
Inputs	Beds	205	209	15	843
	Medical staff	111	173	5	1021
	Non-medical staff	321	256	6	1049

**Table 3:** Main descriptive statistics for different financial systems.

Variables		SSF		MoH	
		Mean	SD	Mean	SD
Outputs	Discharges	6178	6183	11,593	11,464
	Emergencies	43,623	43,638	60,684	60,486
Inputs	Beds	175	176	234	230
	Medical staff	131	131	92	90
	Non-medical staff	338	341	303	302

**Table 4:** Descriptive statistics of the productivity index and its components (2005–2015).

	MPI	EC	PEC	SEC	TC	PTC	STC
**Mean**	1.0253	1.0054	1.0144	0.9993	1.0242	1.0111	1.0300
**SD**	0.0400	0.0253	0.0339	0.0142	0.0220	0.0330	0.0407
**Min**	0.9788	0.9718	0.9769	0.9648	0.9904	0.9634	0.9775
**Max**	1.1341	1.0770	1.1259	1.0319	1.0924	1.0863	1.1527

Note: MPI:Malmquist Productivity Index; EC: Efficiency Change; PEC: Pure Efficiency Change; SEC: Scale Efficiency Change; TC: Technological Change; PTC: Pure Technological Change; STC: Scale Technological Change.

**Table 5:** Mean 95% confidence intervals for MI, EC, and TC for bootstrap sample.

	MPI	EC	TC
**Mean**	1.0253	1.0054	1.0242
**Lower bound**	0.9911	0.8223	0.8534
**Upper bound**	1.0704	1.2133	1.2613

**Table 6:** Differences in descriptive statistics by type of management (2005–2015).

Management System		MPI	EC	PEC.	SEC	TC	PTC	STC
**SSF**	**Mean**	1.0365	1.0093	1.0175	0.9993	1.0365	1.0208	1.0348
	**SD**	0.0470	0.0208	0.0279	0.0081	0.0281	0.0348	0.0469
	**Min**	0.9836	0.9732	0.9769	0.9858	0.9904	0.9767	0.9869
	**Max**	1.0924	1.0489	1.0685	1.0154	1.0924	1.0863	1.1227
**MoH**	**Mean**	1.0141	1.0015	1.0028	0.9997	1.0185	1.0006	1.0248
	**SD**	0.0297	0.0296	0.0164	0.0192	0.0127	0.0291	0.0341
	**Min**	0.9788	0.9718	0.9833	0.9648	0.9967	0.9634	0.9775
	**Max**	1.0860	1.0770	1.0473	1.0319	1.0412	1.0451	1.0850

**Table 7:** Mean 95% confidence intervals for MI, EC, and TC by type of management.

Management System		MPI	EC	TC
**SSF**	**Mean**	1.0365	1.0093	1.0298
	**Lower bound**	1.0008	0.8262	0.8473
	**Upper bound**	1.0919	1.2359	1.2749
**MoH**	**Mean**	1.0141	1.0015	1.0185
	**Lower bound**	0.9827	0.8185	0.8595
	**Upper bound**	1.0489	1.1908	1.2477

**Table 8:** Mean values of MPI and their components for each hospital (2005–2015).

System	Hospital	MPI	Lower Bound	Upper Bound	EC	Lower Bound	Upper Bound	TC	Lower Bound	Upper Bound
SSF	Hospital de Especialidades Pediatrica Omar Torrijos Herrera	1.1341	1.0525	1.3378	1.0489	0.7875	1.4774	1.0590	0.8193	1.3920
SSF	Hospital de Almirante	1.0924	1.0721	1.1133	1.0000	0.7813	1.2900	1.0924	0.8575	1.4002
MoH	Hospital Regional de Azuero Anita Moreno	1.0860	1.0309	1.1332	1.0770	0.8754	1.2558	1.0070	0.8695	1.2469
SSF	Hospital de Changuinola	10807	1.0387	1.1427	1.0375	0.9942	1.0731	1.0151	0.8822	1.2209
MoH	Hospital San Miguel Arcangel	1.0426	1.0243	1.0601	1.0319	0.8656	1.1891	1.0148	0.8790	1.2051
SSF	Policlinica Especializada Dr. Horacio Diaz Gomez	1.0326	0.9966	1.0830	1.0000	0.7859	1.3207	1.0326	0.7965	1.3253
SSF	Hospital Dr. Gustavo Nelson Collado (Chitre)	1.0290	1.0073	1.0717	1.0343	0.8331	1.2311	1.0177	0.8558	1.2616
SSF	Hospital Regional Dr. Rafael Hernandez	1.0265	1.0067	1.0651	1.0161	0.8535	1.2019	1.0210	0.8669	1.2161
SSF	Complejo Hospitalario Metropolitano Arnulfo A. Madrid	1.0227	1.0094	1.0399	0.9946	0.7997	1.2162	1.0331	0.8463	1.2863
MoH	Hospital Materno Infantil Jose Domingo de Obaldia	1.0217	0.9740	1.0566	0.9862	0.8196	1.1538	1.0412	0.8823	1.2672
MoH	Hospital San José de La Palma	1.0185	0.9798	1.0487	1.0020	0.8368	1.1556	1.0190	0.8798	1.2218
MoH	Hospital Luis Chicho Fabrega	1.0135	0.9887	1.0386	1.0000	0.7893	1.2282	1.0135	0.8351	1.2831
SSF	Hospital Dr. Rafael Estevez	1.0134	0.9580	1.0657	1.0117	0.8614	1.1533	1.0188	0.8850	1.1833
MoH	Hospital Rafael H. Moreno	1.0116	0.9946	1.0389	1.0000	0.7903	1.2250	1.0116	0.8320	1.2863
MoH	Hospital Santo Tomas	1.0026	1.0001	1.0284	0.9912	0.8148	1.1789	1.0282	0.8607	1.2569
MoH	Hospital Del Niño	1.0016	0.9896	1.0330	0.9755	0.8283	1.1551	1.0353	0.8758	1.2312
SSF	Hospital Dra. Susana Jones Cano	0.9959	0.9717	1.0182	1.0000	0.7776	1.2198	0.9959	0.8245	1.2908
SSF	Hospital de Chiriqui Grande	0.9904	0.9582	1.0350	1.0000	0.8031	1.2496	0.9904	0.8130	1.2341
MoH	Hospital Dr. Aquilino Tejeira	0.9895	0.9617	1.0232	0.9933	0.7743	1.2082	0.9967	0.8260	1.2928
MoH	Hospital Dr. Joaquin Pablo Franco	0.9893	0.9308	1.0350	0.9876	0.8026	1.1788	1.0148	0.8569	1.2199
SSF	Hospital Ezequiel Abadia	0.9836	0.9247	1.0390	0.9732	0.8108	1.1618	1.0368	0.8733	1.2133
MoH	Hospital Dr. Cecilio A. Castillero	0.9788	0.9351	1.0418	0.9718	0.8062	1.1703	1.0219	0.8571	1.2131

## Data Availability

The data used in this study are not available in any public archive that can be accessed.

## Appendix A

**Table A1:** Hospitals included in the sample and their main characteristics.

Hospital	Network	Province	District	Level of complexity	Area of Influence
Hospital Santo Tomas	MoH	Panamá	Panamá	III	Regional
Hospital Del Niño	MoH	Panamá	Panamá	III	Urban
Hospital de Especialidades Pediátricas Omar Torrijos Herrera	SSF	Panamá	Panamá	III	Rural
Complejo Hospitalario Dr. Arnulfo Arias Madrid	SSF	Panamá	Panamá	III	Urban
Hospital Dra. Susana Jones Cano	SSF	Panamá	San Miguelito	II	Urban
Hospital San Miguel Arcangel	MoH	Panamá	San Miguelito	II	Urban
Hospital de Changuinola	SSF	Bocas del Toro	Changuinola	II	Rural
Hospital de Almirante	SSF	Bocas del Toro	Changuinola	I-II	Rural
Hospital de Chiriquí Grande	SSF	Bocas del Toro	Chiriquí Grande	I-II	Rural
Hospital Regional Dr. Rafael Hernandez	SSF	Chiriquí	David	II	Regional
Hospital Dr. Cecilio A. Castillero	MoH	Herrera	Chitre	II	Urban
Hospital Ezequiel Abadia	SSF	Veraguas	Sona	I-II	Urban
Policlinica Especializada Dr. Horacio Diaz Gomez	SSF	Veraguas	Santiago	II	Urban
Hospital Dr. Rafael Estevez	SSF	Coclé	Aguadulce	II	Urban
Hospital Dr. Aquilino Tejeira	MoH	Coclé	Penonomé	II	Regional
Hospital San José de la Palma	MoH	Darién	Chepigana	I-II	Urban
Hospital Regional de Azuero Anita Moreno	MoH	Los Santos	La Villa de Los Santos	II	Regional
Hospital Rafael H. Moreno	MoH	Los Santos	Macaracas	II	Rural
Hospital Dr. Gustavo Nelson Collado	SSF	Herrera	Chitre	II	Regional
Hospital Luis Chicho Fabrega	MoH	Veraguas	Santiago	II	Regional
Hospital Materno Infantil Jose Domingo de Obaldia	MoH	Chiriquí	David	III	Regional
Hospital Dr. Joaquin Pablo Franco Sayas	MoH	Los Santos	Las Tablas	II	Regional

**Table A2:** Descriptive statistics of the Malmquist index and its components by year.

**Malmquist**	**2005–2006**	**2006–2007**	**2007–2008**	**2008–2009**	**2009–2010**	**2010–2011**	**2011–2012**	**2012–2013**	**2013–2014**	**2014–2015**
Mean	**0.9784**	**0.9708**	**1.0552**	**1.0077**	**0.9419**	**1.0689**	**1.0525**	**1.0151**	**1.1593**	**1.1396**
SD	0.0865	0.1196	0.1000	0.0975	0.1175	0.1517	0.1287	0.1288	0.6770	0.3216
Min	0.8506	0.6483	0.8891	0.8661	0.6286	0.8409	0.6621	0.7634	0.8750	0.8842
Max	1.2196	1.2841	1.2466	1.3412	1.1487	1.5448	1.3335	1.4485	1.5645	1.3565
Efficiency	**2005–2006**	**2006–2007**	**2007–2008**	**2008–2009**	**2009–2010**	**2010–2011**	**2011–2012**	**2012–2013**	**2013–2014**	**2014–2015**
Mean	**1.0640**	**0.9844**	**0.9956**	**1.0117**	**0.9849**	**0.8969**	**1.0125**	**0.9968**	**1.0372**	**1.1280**
SD	0.0932	0.1022	0.1074	0.0795	0.1119	0.1469	0.1181	0.0689	0.1555	0.2192
Min	0.9471	0.6646	0.6940	0.8315	0.6351	0.6472	0.8352	0.8092	0.9169	0.8888
Max	1.2927	1.1408	1.2491	1.1659	1.1710	1.0971	1.3662	1.1124	1.3598	1.2809
Pure Efficiency	**2005–2006**	**2006–2007**	**2007–2008**	**2008–2009**	**2009–2010**	**2010–2011**	**2011–2012**	**2012–2013**	**2013–2014**	**2014–2015**
Mean	**1.0230**	**0.9866**	**1.0181**	**0.9804**	**0.9863**	**1.0279**	**1.0056**	**0.9995**	**1.0326**	**1.0842**
SD	0.0666	0.0714	0.1048	0.0768	0.0764	0.1808	0.1094	0.0791	0.1475	0.1912
Min	0.9193	0.8026	0.8864	0.7358	0.7747	0.7454	0.6993	0.8053	0.8823	0.8747
Max	1.2434	1.1384	1.3590	1.1467	1.1138	1.7628	1.2668	1.2331	1.4173	1.4419
Technological Change	**2005–2006**	**2006–2007**	**2007–2008**	**2008–2009**	**2009–2010**	**2010–2011**	**2011–2012**	**2012–2013**	**2013–2014**	**2014–2015**
Mean	**0.9208**	**0.9852**	**1.0672**	**0.9988**	**0.9579**	**1.2137**	**1.0452**	**1.0189**	**1.0771**	**1.0068**
SD	0.0493	0.0420	0.1204	0.0950	0.0683	0.2001	0.1214	0.1145	0.3261	0.1647
Min	0.7878	0.9348	0.8891	0.9112	0.8028	0.8409	0.6621	0.8758	0.9183	0.8814
Max	0.9994	1.1514	1.4928	1.3412	1.0576	1.5554	1.2249	1.4485	1.5091	1.5310
Pure Technological Change	**2005–2006**	**2006–2007**	**2007–2008**	**2008–2009**	**2009–2010**	**2010–2011**	**2011–2012**	**2012–2013**	**2013–2014**	**2014–2015**
Mean	**0.9211**	**0.9912**	**1.0477**	**1.0417**	**0.9514**	**1.0633**	**0.9984**	**1.1123**	**1.0364**	**1.0275**
SD	0.3360	0.3230	0.3289	0.4092	0.3038	0.3489	0.3284	0.4973	0.4463	0.3405
Min	0.7515	0.7000	0.8118	0.7431	0.7280	0.6344	0.4322	0.8849	0.6898	0.8837
Max	1.0615	1.4376	1.2249	1.6317	1.2268	1.3814	1.2116	1.4148	1.4336	1.5828
Scale Efficiency	**2005–2006**	**2006–2007**	**2007–2008**	**2008–2009**	**2009–2010**	**2010–2011**	**2011–2012**	**2012–2013**	**2013–2014**	**2014–2015**
Mean	**1.0431**	**0.9973**	**0.9852**	**1.0358**	**0.9968**	**0.8842**	**1.0090**	**1.0002**	**1.0044**	**1.0394**
SD	0.1070	0.0761	0.1253	0.0928	0.0656	0.1509	0.0716	0.0654	0.0339	0.0579
Min	0.9609	0.7781	0.6588	0.8315	0.8198	0.5538	0.8697	0.7531	0.9643	0.9913
Max	1.3722	1.1282	1.2257	1.3338	1.1230	1.0934	1.2056	1.1103	1.0999	1.2049
Scale Technological Change	**2005–2006**	**2006–2007**	**2007–2008**	**2008–2009**	**2009–2010**	**2010–2011**	**2011–2012**	**2012–2013**	**2013–2014**	**2014–2015**
Mean	**1.0061**	**1.0090**	**1.0334**	**0.9847**	**1.0179**	**1.1465**	**1.0613**	**0.9461**	**1.0528**	**0.9820**
SD	0.3696	0.3196	0.3305	0.3617	0.3180	0.3875	0.3354	0.3465	0.3212	0.2944
Min	0.7430	0.8010	0.9042	0.8058	0.7839	0.8903	0.9309	0.5998	0.9570	0.8661
Max	1.2555	1.3861	1.5046	1.2963	1.2737	1.5714	1.5320	1.0850	1.3818	1.0816
